# What competencies do environmental health graduates need to manage social determinants of health?

**DOI:** 10.1186/s12199-021-01036-x

**Published:** 2021-12-04

**Authors:** Athar Omid, Fateme Sepyani, Nikoo Yamani, Hamidreza Pourzamani, Pejman Aghdak

**Affiliations:** 1 Department of Medical Education, Medical Education Research Center, Isfahan University of Medical Sciences, Isfahan, Iran; 2 Student Research Committee, School of Health, Isfahan University of Medical Sciences, Isfahan, Iran; 3 Department of Environmental Health Engineering, School of Health, Isfahan University of Medical Sciences, Isfahan, Iran

**Keywords:** Competencies of Bachelor of Environmental Health Engineering students, Social Determinants of Health

## Abstract

**Background:**

Graduates of environmental health engineering should be able to manage Social Determinants of Health (SDH) and acquire the essential competencies during their studies at university. This study was performed to determine the expected competencies of environmental health graduates in a way to be able to manage environmental and Social Determinants of Health according to their job description.

**Methods:**

This descriptive cross-sectional study was performed using Delphi technique. First, the literature review was done and the Delphi technique was performed in three rounds. The purposeful sampling was used and 50 people were selected among the specialists in the field of environmental health engineering and SDH. Participants answered an open-ended question, for the first round. Then, a questionnaire with 8 areas was designed based on the results of the first round and distributed for the second round. Data analysis was performed using descriptive statistics. The third round was done to reach the agreement on final items.

**Results:**

The agreement on the items of the third round of Delphi was more than 70%. The final results showed eight competency areas under which 29 competencies were defined. Competency areas included expert knowledge, reasoning and planning, advocacy, system-based practice, professionalism, instructional expertise, social and personal skills and, research and self-development. The first three priorities of the required competency areas were expert knowledge (4.46 ± 0.55), professionalism (4.42 ± 0.64), and advocacy (4.32 ± 0.77).

**Conclusions:**

It is necessary that environmental health engineers achieve necessary competencies regarding managing SDH, upon their graduation. It is suggested to integrate these competencies into the curriculum of environmental and health engineering in Iran universities.

## Background

The definitive role of Social and Environmental Determinants of Health (SDH) has long been recognized and considered by the World Health Organization. According to the definitions of WHO, SDH are “the conditions in which people are born, grow, work, live, and age, and, the wider set of forces and systems shaping the conditions of daily life”. In fact, these factors play the most important role in human health. These factors include unemployment and job security, nutrition and food security, healthy lifestyle, education, housing, and environment [1].

Bachelor of environmental health engineering is one of the disciplines in Iran Universities of Medical Sciences with the aim of promoting community health. Graduates of this discipline have a duty to manage as much as possible the SDH which are related to their field [2]. They supervise and manage water, sewage, air, and soil pollution as well as radiation, food, housing, and public places hygiene. They also learn to face a number of problems, such as water and food problems, and inadequate environmental health standards in the field of health. So, in order to manage these duties and tasks [3, 4], they must have acquired the necessary competencies during their studies at university. Accordingly, in their curriculum, the role of SDH and the necessary competencies to manage these factors should be considered.

Bachelor of Environmental Health Engineering is presented during 8 semesters and the main courses include solid waste management (general course), water treatment, wastewater treatment, air pollution and food hygiene. Some complementary courses such as environmental chemistry, water and wastewater microbiology, fluid mechanics, and, physical, and chemical processes of water and wastewater are also taught. Moreover, they pass 16 credit internship, as their final course. Considering the fact that the graduates are supposed to work at health centers, the theoretical courses are not corresponding to their duties. Also, needs assessment studies in the field of environmental health engineering show that the current curriculum does not correspond to their job responsibilities. In these studies, it has been suggested that the curriculum of environmental health engineering be carefully revised and that the educational content be adapted to their job responsibilities and educational needs [5–8]. In addition, it has been suggested that on-the-job training for them be designed to meet these needs and thus improve the quality of service delivery [9]. This mismatch has left a big percentage of graduates unemployed [10] and has also made students’ attitudes toward their field of study and their future careers unfavorable [11]. Therefore, it is necessary to re-evaluate the needs and revise the curriculum of this discipline. This is the first step in curriculum planning and in fact an important factor in ensuring the effectiveness of educational programs [12]. Therefore, this study was conducted to determine the expected job competencies of environmental health engineers in a way to enable them to manage SDH, according to their job description, and, to provide the needed suggestions for revising the objectives and content of the environmental health engineering curriculum.

## Methods

This was a descriptive cross sectional study which was performed in two parts in 2019. The first part was literature review and the second part was done through Delphi technique. Literature review was conducted to determine the competencies needed to manage SDH. The databases (ISI Web of Sciences, PubMed, Scopus, ERIC) and E journal of Magiran and SID (Persian databases) with the keywords of “social determinants of health”, “environmental health engineering” and “competency”, both in English and Persian were searched. The results (related articles) were carefully studied and a list of SDH, related to the job description of environmental health engineers as well as the necessary competencies to manage these factors were itemized.

The second part of the study was done as a Delphi with three rounds. The purposive sampling using snowball technique was used and 50 people were selected among the specialists in the field of environmental health engineering and SDH, working in health centers. Inclusion criteria were having at least 1 year of work experience and willingness to participate in the study, and if they refused to continue participation and were out of access, they were excluded from the study.

### First round of Delphi

In the first round of Delphi, a question tailored to the needs assessment was designed. The question was, “What competencies should environmental health engineers have to manage the social and environmental determinants of health?”

This question was distributed among the participants through face-to-face visits or by e-mail. Before distributing the questionnaire, an informed consent to participate in the study was obtained from participants in person or by phone. After 2 to 4 weeks, the answer sheets were collected. Responses were analyzed and similar and duplicate competencies were removed.

### Second round of Delphi

In this round, first, the list earned from the first round of Delphi was merged with the list obtained from the literature review and in total, 29 competencies were listed. These 29 competencies were then categorized into 8 competency areas by the research team. Then, the competency statements were designed as a questionnaire. A five-point scale was used as very high, high, medium, low, and very low for the questionnaire. The participants were asked to identify the need for each of these competencies in order to manage SDH by selecting appropriate scale. During the analysis, the responses to high and very high as well as to low and very low, were accumulated in one column to be able to determine the responses below 70 % (Table 2). At the beginning of this questionnaire, the purpose of the study was explained and the demographic information of the participants was asked. The validity of this questionnaire was confirmed by 8 people, including medical education (*n* = 2), social determinants of health (*n* = 2), and environmental health experts (*n* = 4).

The questionnaires were distributed through face-to-face visits or by email among the same group, participating in the first round of Delphi (*n* = 50). The deadline to respond the questionnaires was 2 to 4 weeks and then, follow-up was done by email or phone call.

All questionnaires were analyzed using descriptive statistics (percentage, mean and standard deviation). In this round, except for the two competencies (prioritizing problems related to SDH and studying and doing research in the field of SDH), more than 70% of the participants agreed or strongly agreed that the rest of the items were needed [13].

### Third round of Delphi

Since, there was no agreement on the two mentioned items, a questionnaire including these two items, was prepared and distributed among the same group (*n* = 50). Participants were asked to re-score these items. The results showed that more than 70% of the participants agreed or strongly agreed on each item. Therefore, according to the agreement reached on all items, the Delphi rounds ended [13].

## Results

As the results of literature review, 32 articles were reviewed, of which 12 articles met the inclusion criteria and were related to job competencies of environmental health engineering. So, 30 competencies were extracted from literature.

As the results of Delphi, the demographic information of the participants (*n* = 50) is shown in Table 1. Fifty-six percent of participants had more than 15 years of experience, 62% were undergraduates and 86% worked in the field of environmental health. The participants in this study were the same in all three rounds of Delphi and no one was removed or added.

**Table 1:** Mean and standard deviation of age, and frequency distribution of demographic variables of participants

Item	Mean and standard deviation
Age (year)	41.6 ± 8.4
	Frequency
Sex	
Female	54%%
Male	46%
Work experience (year)	
1–5 years	26%
6–10 years	12%
11–15 years	6%
More than 15 years	56%
Area of expertise	
Environmental health	86%
SDH	14%
Degree	
Bachelor	62%
Master	20%
PhD	14%

The results of the first round of Delphi revealed 29 competencies. Items such as communication skills [14], and advocacy (which means the ability to influence key individuals who can build support for interventions and help to solve problems related to SDH [15]), the ability to use the appropriate technology [5], and the ability to involve people, organizations and institutions [15] were similar to the results of literature review. But, presentation skills, professional ethics, stress and anger management, familiarity with environmental health laws, earned from the first round of Delphi and were added to the results of the literature review.

The 29 competencies were classified into 8 competency areas including expert knowledge, reasoning and planning, advocacy, system-based practice, professionalism, instructional expertise, social and personal skills, and research and self-development.

The results of the second round of Delphi are presented in Table 2. As the table shows, except for the two items of ability to “prioritize problems related to SDH” and “research and follow evidence in the field of SDH”, for the rest of the items, more than 70% of participants identified a very high or high need.

**Table 2:** Frequency, mean, and standard deviation of participants’ answers to each of the questionnaire items

Competency areas	Competencies	Very high + high	Moderate	Very low + low	Mean	SD
Expert knowledge	Having knowledge about health and dimensions of health	**82%**	**18%**	**0%**	**4.38**	**0.78**
	Having knowledge about SDH	**94%**	**4%**	**2%**	**4.5**	**0.68**
	Recognizing SDH related to environmental health	**92%**	**6%**	**2%**	**4.46**	**0.71**
	Recognizing factors affecting SDH related to environmental health	**93.87%**	**4.08%**	**2.04%**	**4.53**	**0.68**
Reasoning and planning	Systematically collecting and organizing information about the state of society in terms of SDH	**74%**	**20%**	**6%**	**4.04**	**0.90**
	Interpreting information and preparing a list of problems related to SDH	**80%**	**18%**	**2%**	**4.20**	**0.81**
	Prioritizing problems related to SDH in society	**68%**	**28%**	**4%**	**3.98%**	**0.83**
	Identifying the effective factors in the occurrence of problems related to SDH in the community	**78%**	**20%**	**2%**	**4.12%**	**0.86**
	Determining the necessary interventions to solve problems related to SDH	**76%**	**18%**	**6%**	**4.14**	**0.94**
	Planning to implement designated interventions to solve problems related to SDH	**82%**	**16%**	**2%**	**4.18**	**0.77**
	Having creativity and initiative in formulating intervention for problems related to SDH	**78%**	**20%**	**2%**	**4.04**	**0.76**
Professionalism	Being sensitive to problems related to SDH	**78%**	**20%**	**2%**	**4.20**	**0.84**
	Having commitment to environmental health laws	**89.79%**	**6.12%**	**4.08%**	**4.42**	**0.87**
	Respecting professional ethics and human rights	**92%**	**8%**	**2%**	**4.55**	**0.71**
	Having Commitment and responsibility to solve problems related to SDH	**85.7%**	**10%**	**4.08%**	**4.44**	**0.85**
System-based practice	Recognizing effective stakeholders, institutions, and all community resources to solve problems related to SDH	**84%**	**16%**	**0%**	**4.20**	**0.7**
	Recognizing and making use of the facilities and capacities of stakeholders, institutions, and all community resources to solve problems related to SDH	**75.5%**	**22.44%**	**2.04%**	**4.12**	**0.83**
	Understanding legal systems and using them to solve problems related to SDH	**76%**	**18%**	**6%**	**4.26**	**1.01**
Advocacy	Engaging and sensitizing stakeholders and effective institutions for problems related to SDH	**86%**	**8%**	**6%**	**4.38**	**0.88**
	Influencing key individuals to build support for intervention to Solve problems related to SDH	**86%**	**8%**	**6%**	**4.26**	**0.85**
Instructional expertise	Using technology and instructional media (use of computers and modern technologies such as movies, etc.)	**84%**	**12%**	**4%**	**4.08**	**0.91**
	Using appropriate instructional methods	**86%**	**8%**	**6%**	**4.10**	**0.94**
	Using appropriate articulation with target group health literacy	**86%**	**8%**	**6%**	**4.27**	**1.00**
Social and personal skills	Working effectively as a member or leader of a health professional team or other professional groups	**80%**	**14%**	**6%**	**4.24**	**0.99**
	Communicating effectively with other health professional and the public	**88%**	**6%**	**6%**	**4.39**	**0.93**
	Controlling strong emotions (anger, stress, etc.)	**84%**	**12%**	**4%**	**4.43**	**0.87**
	Motivating themselves to continuously follow the results of activities	**78%**	**18%**	**4%**	**4.08**	**0.83**
Research and self-development	Doing research and following evidence in the field of SDH	**65.3%**	**30.61%**	**4.08%**	**3.94**	**0.97**
	Evaluating the impact of interventions to solve problems related SDH	**82%**	**%14**	**4%**	**4.14**	**0.82**

In the third round of Delphi, these two items (to “prioritize problems related to SDH” and “Research and follow evidence in the field of SDH”) were questioned in the form of a questionnaire, and were identified by more than 70% of the participants as having very high and high needs. As Table 3 shows, the first three priorities of competency areas were expert knowledge, professionalism, and advocacy with mean and standard deviation of 4.46 ± 0.55, 4.42 ± 0.64, and 4.32 ± 0.77, respectively.

**Table 3:** Mean and standard deviation of participants’ response to each of the competency areas

	Expert knowledge	Reasoning and planning	Advocacy	System-based practice	Professionalism	Instructional expertise	Social and personal skills	Research and self-development
**Mean**	**4.46**	**4.17**	**4.32**	**4.19**	**4.42**	**4.15**	**4.29**	**4.15**
**SD**	**.55**	**.73**	**.77**	**.68**	**.64**	**.81**	**.71**	**.59**

## Discussion

This study was performed to determine the expected competencies of environmental health graduates in a way to be able to manage environmental and Social Determinants of Health. The results showed eight competency areas and 29 competencies. The competency areas were expert knowledge, reasoning and planning, advocacy, system-based practice, professionalism, instructional expertise, social and personal skills, and research expertise which are necessary for training environmental health experts.

Among determined priorities in competency areas, were advocacy, personal and social skills, and system-based practice. These results cohere with the findings of some previous researches. Marandi and Vahidi showed that these competencies were necessary for having organizations’ and institutions’ support and participation to control Social Determinants of Health [16, 17]. Alami, sited from final report of SDH commission of World Health Organization (2008), had pointed to participation of organizations who were effective on SDH, in health sector activities [18]. In their study with the title “a review on 3-year performance of health and food safety councils”, Damary and colleagues have mentioned that improving health indices in national and provincial level needed inter-sectional cooperation and people’s participation. Also, according to their investigation, informing stake holders and people of the society as well as using advocacy technique for developing inter-sectional cooperation was important. Moreover, they have mentioned that empowering faculty members for improving their attitude and skills towards SDH approaches were important activities [15]. Likewise, in DeVoe and colleagues’ study about primary health vision for integrating SDH, they have pointed to cooperation between different organizations, standardizing the data collection and presentation, and participation in health activities of society as important tasks [19]. In fact, the responsibility of providing society’s health is much more than what Ministry of Health can provide by itself and needs inter-sectional coordination and cooperation and using all their capacities.

Wartman and colleagues, in their study about the role of university health centers in addressing social responsibilities, have paid attention to issues such as creating inter-disciplinary relations, making interaction between professions and disciplines, and developing multidisciplinary groups [20] which matches the results of the present study. In another study in Swaziland University regarding developing graduate studies, they have determined the needs of stake holders and organizations regarding environmental health, and the results have emphasized on practical skills, research capabilities, project management, entrepreneurship skills, advanced lab analysis skills, and computer skills, which mainly corresponds to the results of the present study [21]. Most of these studies also show, in addition to advocacy and system-based practice, there is a need to personal, social and research capabilities in order to promote society’s health and manage SDH.

The present study also emphasized on reasoning and planning as needed capabilities. The result of Grimm and colleagues’ study in Nebraska University in 2012 showed that cultural capabilities and communication skills were two important competencies needed in public health. Their results, also revealed that the most emphasized educational needs were financial planning, managerial skills, and analytical/assessment skills [14]. These results are almost the same as the results of the present study especially in the fields of communication skills, reasoning, and planning. In another study by Hamdi and colleagues about Job analysis of environmental health staff, some tasks such as health education, assessment of health services provided in health networks, being familiar with environmental health regulations, and computer software have been mentioned as educational needs [5]. These educational needs are mostly in areas of education, reasoning and planning, professionalism, and system-based practice which correspond to present study.

The review of other studies showed that each study had pointed to some of the competencies needed to manage Social Determinants of Health. While the present study tried to determine most of the needed competencies for managing SDH, in the context of Iran. Although, we tried to encompass a comprehensive view toward responding the research question using literature review, one of the limitations of this study was not using the views of more faculty members and experts other universities of Iran. But the strength of this study was suing the views of faculty members and experts in the field of Social Determinants of Health as well as environmental health staff which gave us a vast range of all stakeholders’ views.

## Conclusion

Considering the tasks of environmental health staff in managing environmental and Social Determinants of Health such as water, foods, habitation, etc., it is necessary that these staff achieve necessary competencies upon their graduation (Table 2) in competency area such as advocacy, reasoning and planning, instructional techniques, personal and social skills, and professionalism. Therefore, it is suggested to develop a competency-based curriculum for this field taking advantage of the competencies earned as a result of the present study.

## Data Availability

The datasets used and/or analyzed during the current study available from the corresponding author on reasonable request.
